# Development and internal validation of a risk prediction model for ipsilateral upper-limb lymphedema following breast cancer surgery

**DOI:** 10.3389/fonc.2026.1823165

**Published:** 2026-06-03

**Authors:** Yufei Fan, Gaofeng Yang, Yumeng Zeng, Yanfei Lu, Fan Feng, Xiudi Wang

**Affiliations:** 1 Department of Gynecology, Hangzhou Women’s Hospital, Hangzhou, Zhejiang, China; 2 Department of General Surgery, The Third Affiliated Hospital of Chongqing Medical University, Chongqing, China; 3 Department of Breast Surgery, Hangzhou Women’s Hospital, Hangzhou, Zhejiang, China; 4 Physical Examination Center, Hangzhou Women’s Hospital, Hangzhou, Zhejiang, China

**Keywords:** breast cancer, lymphedema, nomogram, pectoral nodes dissection, risk factors

## Abstract

**Background:**

Accurate prediction of breast cancer-related lymphedema (BCRL) is essential for identifying high-risk patients, guiding early preventive interventions, and improving postoperative quality of life among breast cancer survivors. This study aimed to develop and internally validate a clinically practical predictive nomogram for BCRL.

**Methods:**

This retrospective cohort study included 234 patients undergoing breast cancer surgery, of whom 27 (11.5%) developed BCRL. Candidate predictors were first screened using LASSO-based regression. Subsequently, clinically relevant variables were entered into multivariable analysis, and Firth penalized logistic regression was applied to reduce sparse-data bias and improve estimate stability in the setting of limited outcome events. A nomogram was then constructed based on the statistically significant predictors. Model performance was evaluated by receiver operating characteristic (ROC) analysis, calibration measures, bootstrap internal validation, and decision curve analysis (DCA).

**Results:**

The final nomogram incorporated four variables: surgery type, pectoral nodes dissection, number of harvested lymph nodes, and N stage. In the Firth penalized logistic regression model, breast-conserving surgery was associated with a lower risk of BCRL, whereas pectoral nodes dissection, higher N stage, and a greater number of harvested lymph nodes were associated with increased risk. The model demonstrated excellent discriminative ability, with an apparent area under the curve (AUC) of 0.964 and a bootstrap optimism-corrected AUC of 0.954. Calibration analysis showed a calibration-in-the-large of 0.000, an apparent calibration slope of 1.000, and an optimism-corrected calibration slope of 0.711, indicating residual overfitting. The apparent Brier score was 0.0469, with an optimism-corrected value of 0.0537. DCA showed that the nomogram provided favorable net benefit relative to the treat-all and treat-none strategies over much of the clinically relevant threshold range (5%–80%).

**Conclusion:**

We developed and internally validated a clinically practical nomogram for predicting BCRL using four readily available variables. The model showed strong discrimination, acceptable calibration, and potential clinical utility. Pending external validation, this tool should be regarded as a supportive risk-stratification aid rather than a stand-alone clinical decision-making instrument, and it may help identify patients who warrant closer surveillance, preventive counseling, or early rehabilitation referral.

## 1 Introduction

Breast cancer survivorship has improved substantially, shifting clinical focus toward long-term treatment-related morbidity. One of the most frequent and distressing complications is breast cancer–related lymphedema (BCRL) of the ipsilateral upper limb, which can manifest as swelling, heaviness, pain, functional limitation, recurrent infection (e.g., cellulitis/lymphangitis), and persistent psychosocial burden. Importantly, BCRL is not merely a cosmetic issue: population-based evidence indicates that patients with BCRL experience significantly worse health-related quality of life (HRQoL) across multiple physical and psychosocial domains compared with patients without BCRL, and these impairments can persist for years after treatment (1).

The reported incidence of BCRL varies widely due to differences in case definitions, diagnostic methods (e.g., circumference/volume, bioimpedance, clinical diagnosis), and follow-up duration. Nevertheless, high-quality evidence consistently suggests that BCRL affects a substantial proportion of survivors. In a landmark systematic review and meta-analysis, the pooled incidence of unilateral arm lymphedema was approximately one in five when restricted to prospective cohort data, with higher risk observed after more extensive axillary surgery and in those who are overweight or obese. Notably, the incidence appeared to rise during the first two postoperative years, underscoring the clinical importance of early surveillance windows (2).

From a pathophysiological perspective, BCRL reflects impaired lymphatic transport capacity following lymphatic injury or obstruction from axillary surgery and/or regional irradiation, leading to progressive fluid accumulation and, over time, tissue remodeling with fibrosis and adipose deposition. The International Society of Lymphology (ISL) emphasizes that lymphedema may be acute, transitory, or chronic, and typically requires long-term management once established. The ISL staging framework also recognizes a latent or subclinical phase (Stage 0), during which lymphatic transport is impaired but overt swelling may not yet be evident—highlighting the opportunity for early identification before irreversible changes occur (3).

Clinically, the timing of BCRL onset supports focusing on early postoperative prediction. Longitudinal survivorship data indicate that a large proportion of cases occur relatively soon after breast cancer diagnosis/treatment; in one cohort, most lymphedema cases developed within the first two years. Similarly, in a large prospective survivorship study (Pathways Study), the mean time to BCRL diagnosis was within the first year, with the observed range extending into the second postoperative year. Together, these data support the pragmatic selection of a 2-year postoperative endpoint for risk stratification and early preventive interventions (4).

Multiple treatment-related and patient-related factors have been associated with BCRL, including axillary lymph node dissection (ALND), greater number of nodes removed, regional nodal irradiation, higher body mass index (BMI), and other clinical and perioperative characteristics. Recent evidence syntheses continue to affirm that these associations are broadly consistent, while also highlighting that the overall quality of evidence across risk-factor meta-analyses is often limited by bias and heterogeneity. Consequently, there remains a need for well-designed, contemporary datasets with standardized outcome definitions and robust modeling approaches to support individualized prediction and targeted prevention in real-world settings (5).

Although many prediction models and nomograms for BCRL have been published, their clinical utility remains uncertain. A systematic review and critical appraisal of BCRL prediction models found that, despite apparently moderate-to-high discrimination in some studies, most models were judged to have a high risk of bias, with limited external validation and substantial variability in predictor selection and outcome ascertainment. These limitations have hindered broader implementation across clinical settings and populations. Therefore, there remains a need for clinically interpretable prediction models developed using standardized outcome definitions, transparent modeling strategies, and rigorous internal validation. Developing and validating a model using local institutional data may provide meaningful incremental value, particularly if the resulting tool is tailored to local practice patterns and supports a feasible postoperative surveillance strategy within the first two years (6, 7).

Accordingly, the aims of the present study were to (1) identify independent risk factors for ipsilateral upper-limb BCRL within two years after breast cancer surgery using data from Hangzhou Women’s Hospital, and (2) develop and internally validate an individualized prediction model to estimate each patient’s 2-year risk of BCRL, thereby enabling risk-stratified follow-up and preventive interventions in routine clinical practice.

## 2 Methods

### 2.1 Study design and data sources

This was a single-center, retrospective cohort study conducted at the Breast Surgery Department of Hangzhou Women’s Hospital. Eligible patients were identified through the hospital electronic medical record (EMR) system. Clinical information was extracted from multiple institutional data sources, including the electronic medical record system, operative and anesthesia records, pathology reports, radiotherapy records, systemic therapy records (chemotherapy, targeted therapy, and endocrine therapy), and follow-up documentation from outpatient clinics and rehabilitation services. The study period spanned from 01/01/2014 to 02/28/2026. Data extraction and curation were performed using a prespecified case report form, and variables were harmonized across data sources to ensure consistency. This study is reported in accordance with the STROBE statement for cohort studies and the TRIPOD statement for prediction model studies. The study protocol was reviewed and approved by the Ethics Committee of Hangzhou Women’s Hospital (Approval No (2026).: Medical Ethics Review A No. 004). Given the retrospective nature of the study and the use of de-identified data, the requirement for informed consent was waived in accordance with institutional policies and applicable regulations. The study was conducted in accordance with the Declaration of Helsinki.

### 2.2 Study population

Women with pathologically confirmed breast cancer who underwent breast surgery with or without axillary intervention were screened for inclusion. Patients were eligible if they had (1) a diagnosis of primary breast cancer, (2) definitive breast surgery (breast-conserving surgery or mastectomy) with or without sentinel lymph node biopsy (SLNB) and/or axillary lymph node dissection (ALND), and (3) availability of postoperative follow-up data sufficient for ascertainment of BCRL, including at least one documented postoperative follow-up visit and/or postoperative upper-limb measurement record. Exclusion criteria included (1) pre-existing ipsilateral upper-limb edema or severe upper-limb conditions that could confound lymphedema assessment (e.g., major trauma, advanced inflammatory or vascular disease), (2) synchronous bilateral breast cancer treated with bilateral surgery, (3) follow-up duration < 6 months without documented BCRL outcome ascertainment, and (4) transfer of care to another institution resulting in missing key treatment information or inability to ascertain the primary outcome.

### 2.3 Outcome definition and measurement

The primary outcome was the development of clinically manifest breast cancer-related lymphedema (BCRL) within 2 years postoperatively. Upper limb circumferences had been routinely measured during postoperative follow-up by trained personnel using a standardized, flexible non-stretch tape, and these data were retrospectively retrieved for the present study. Measurements were taken at fixed 4-cm intervals, starting from the ulnar styloid process proximally to the axillary fold. Total limb volume was subsequently calculated using the truncated-cone (frustum) formula. BCRL was defined as a Relative Volume Change (RVC) of ≥10% compared to baseline. To exclude the interference of transient postoperative edema, all patients included in the BCRL group underwent a minimum of 3 months of continuous observation following initial diagnosis to confirm the non-transitory nature of the lymphedema. The RVC metric was utilized to account for baseline asymmetry and non-lymphedema-related systemic weight changes over time.

#### 2.3.1 Limb volume calculation (truncated-cone method)

Arm volume was calculated from serial circumference measurements using the truncated-cone (frustum) method. For each adjacent pair of circumference measurements (*C1* and *C2*) separated by segment length *h* (here, h = 4 cm), segment volume was computed as:


$$V_{\text{segment}}=\frac{h}{12\pi }(C_{1}^{2}+C_{1}C_{2}+C_{2}^{2})$$

Total limb volume was obtained by summing the volumes of all segments from wrist to the proximal measurement point:


$$V_{\text{arm}}=\sum V_{\text{segment}}$$

Volumes were calculated separately for the affected (ipsilateral) arm and the unaffected (contralateral) arm at each time point.

#### 2.3.2 Relative volume change

Relative Volume Change (RVC) was calculated to account for baseline asymmetry and non-lymphedema–related changes in limb size over time (e.g., weight change), using the following formula:


$$\text{RVC}=\frac{A_{2}\times U_{1}}{U_{2}\times A_{1}}-1$$

where *A1* and *A2* denote the affected-arm volumes at baseline and follow-up, and *U1* and *U2* denote the unaffected-arm volumes at baseline and follow-up, respectively.

Baseline volume (*A1* and *U1*) was defined as the preoperative measurement when available; otherwise, it was defined as the earliest postoperative measurement prior to 3 months that was documented before any outcome event ascertainment.

### 2.4 Data collection and variables

Extracted variables included demographic characteristics (age, Body Mass Index (BMI)), tumor characteristics (tumor size, T stage, N stage, hormone receptor (HR) status, HER2 status, Ki-67 index, lymphovascular invasion), and surgical/treatment variables (type of surgery, total number of harvested lymph nodes, number of positive lymph nodes, pectoral (Rotter’s) nodes dissection, surgery time, total drainage volume, and adjuvant therapy details). Overall receipt of radiotherapy and chemotherapy was extracted when available. However, detailed treatment parameters were not consistently recorded in a standardized format across the full study period. Specifically, granular radiotherapy information, including regional nodal irradiation fields, dose-volume parameters, fractionation schedules, and boost details, as well as regimen-specific chemotherapy information, including taxane exposure, cumulative dose, number of cycles, and treatment sequencing, were incompletely or heterogeneously documented in the retrospective records. To avoid substantial misclassification, sparse-data bias, and unstable coefficient estimation, these detailed treatment parameters were not included as candidate predictors in the final model. This decision was made for data-quality reasons and does not imply that these variables are clinically unimportant. These variables were considered as candidate predictors for subsequent feature screening and multivariable model development.

Missing data were confined to two continuous variables: total drainage volume and surgery duration. In the primary analysis, missing values were imputed using the median to preserve sample size. In addition, a multiple imputation by chained equations (MICE) sensitivity analysis was performed to evaluate the robustness of the findings under an alternative missing-data strategy. No imputation was required for categorical variables because they were fully observed.

#### 2.4.1 Follow-up schedule and observation window

Patients were followed from the date of surgery until the first occurrence of the relevant outcome, 2 years postoperatively, death, loss to follow-up, or the last documented clinical encounter, whichever came first. In routine practice, limb assessments were typically performed at approximately 3, 6, 12, and 24 months after surgery, with additional assessments as clinically indicated.

To reduce measurement variability, assessors followed a standardized institutional measurement protocol, including predefined anatomical landmarks and identical interval spacing for both upper limbs at each visit.

### 2.5 Statistical analysis and model development

Continuous variables were presented as mean ± standard deviation (SD) or median with interquartile range (IQR), depending on the normality of the distribution. Categorical variables were expressed as frequencies and percentages.

#### 2.5.2 Missing data handling

Missing data were limited to two continuous variables: total drainage volume (34/234, 14.53%) and surgery duration (7/234, 2.99%) (**Supplementary Table 1**). No missing data were present in the outcome variable or in the main clinicopathological predictors included in the final model. In the primary analysis, missing values were imputed using the median, given the limited extent of missingness. To further assess whether the main findings were sensitive to the method of missing-data handling, a sensitivity analysis based on multiple imputation by chained equations (MICE) was performed. Ten imputed datasets were generated using multiple imputation by chained equations. The same regression model was fitted separately in each imputed dataset. Regression coefficients, standard errors, odds ratios, confidence intervals, and P values were then pooled using Rubin’s rules. The multiple-imputation sensitivity analysis was primarily used to assess whether the estimated associations of key predictors were robust to the missing-data handling strategy. Model performance metrics were examined descriptively across imputed datasets where applicable, but the primary inference of this sensitivity analysis was based on the pooled regression estimates rather than on re-derivation of a separate final nomogram.

Differences in baseline characteristics between the lymphedema and non-lymphedema groups were compared using the Student’s t-test or Mann-Whitney U test for continuous variables, and the Pearson’s Chi-square test or Fisher’s exact test for categorical variables. To develop a parsimonious and clinically interpretable prediction model, a two-step feature selection strategy was applied. First, LASSO-based variable screening was performed using L1-penalized logistic regression implemented in the scikit-learn library in Python. The regularization parameter was selected by 5-fold stratified cross-validation from a predefined grid of candidate values, and the optimal value in the final analysis was C = 0.76095. Predictors with non-zero coefficients were retained as candidate variables for subsequent modeling.

To qualitatively assess the robustness of variable selection, we examined whether the predictors identified by LASSO screening were clinically plausible and whether their directions of association were consistent in the subsequent Firth penalized logistic regression model. Predictors related to surgical extent and nodal burden, particularly surgery type, pectoral nodes dissection, number of harvested lymph nodes, and N stage, remained the most clinically interpretable and statistically supported variables for model construction. However, formal stability selection based on repeated subsampling, bootstrapped variable-selection frequencies, or extensive alternative penalty specifications was not performed; therefore, the stability of predictor selection should be interpreted cautiously.

Second, variables identified through LASSO screening, together with clinically relevant predictors, were further evaluated in multivariable analysis. Given the limited number of BCRL events and the concern for sparse-data bias, Firth penalized logistic regression was used to obtain more stable effect estimates. Based on the LASSO results and clinical relevance, six candidate predictors were entered into the Firth model: surgery type, pectoral nodes dissection, number of harvested lymph nodes, N stage, tumor size, and BMI. Detailed radiotherapy and chemotherapy parameters were not entered into the multivariable model because they were not available in a sufficiently standardized and complete form for reliable modeling; their exclusion was therefore recognized *a priori* as a potential source of residual confounding. Predictors that remained statistically significant were subsequently incorporated into the final nomogram. Effect estimates are presented as odds ratios (ORs) with corresponding 95% confidence intervals (CIs).

Model performance was evaluated in terms of discrimination, calibration, and clinical utility. Discrimination was assessed using the area under the receiver operating characteristic curve (AUC). Calibration was assessed by calibration-in-the-large, calibration slope, Brier score, and calibration curve analysis. Internal validation was performed using 1000 bootstrap resamples to estimate optimism and derive optimism-corrected performance measures. Decision curve analysis (DCA) was used to assess potential clinical utility across threshold probabilities ranging from 5% to 80%.

All statistical analyses were conducted using Python software. Data preprocessing and management were performed using pandas, LASSO-based variable screening was implemented using scikit-learn, and regression analyses were conducted using stats models. A two-sided *P* value < 0.05 was considered statistically significant.

## 3 Results

### 3.1 Baseline patient characteristics

A total of 234 patients with breast cancer were enrolled in this study, among whom 27 (11.5%) developed breast cancer-related lymphedema (BCRL) during the follow-up period. Baseline comparisons showed that patients in the BCRL group had significantly larger tumor sizes (39.7 vs. 26.1 mm, *P* = 0.006), a higher number of positive lymph nodes (3.0 vs. 0.8, *P* < 0.001), and a more extensive burden of harvested lymph nodes (16.8 vs. 6.9, *P* < 0.001) compared to those without BCRL. Furthermore, categorical variables including mastectomy, axillary lymph node dissection (ALND), pectoral lymph node dissection, and postoperative radiotherapy were significantly associated with the incidence of BCRL (all *P* < 0.05) (**Table 1**).

**Table 1:** Comparison results of each factor between groups.

Variables	Lymphedema (N = 27)	Non-Lymphedema (N = 207)	P-value
Age (years)	52.9 ± 10.9	51.7 ± 11.2	0.619
BMI (kg/m²)	23.6 ± 1.7	23.6 ± 2.0	0.872
Tumor Size (mm)	39.7 ± 23.3	26.1 ± 14.3	**0.006**
Positive Lymph Nodes (n)	3.0 ± 2.8	0.8 ± 1.2	**<0.001**
Harvested Lymph Nodes (n)	16.8 ± 7.3	6.9 ± 8.5	**<0.001**
Ki-67 Index (%)	18.9 ± 9.9	19.6 ± 9.9	0.724
Surgery Time (min)	159.6 ± 31.6	151.0 ± 33.0	0.196
Total Drainage Volume (ml)	314.9 ± 138.4	266.4 ± 112.9	0.091
Type of Surgery			**0.002**
- Mastectomy	26 (96.3%)	133 (64.3%)	
- Breast-conserving	1 (3.7%)	74 (35.7%)	
Axillary Surgery			**<0.001**
- SLNB	2 (7.4%)	125 (60.4%)	
- ALND	25 (92.6%)	82 (39.6%)	
Pectoral Nodes Dissection			**<0.001**
- No	14 (51.9%)	204 (98.6%)	
- Yes	13 (48.1%)	3 (1.4%)	
Chemotherapy			0.457
- No	0 (0.0%)	11 (5.3%)	
- Yes	27 (100.0%)	196 (94.7%)	
Radiotherapy			**<0.001**
- No	2 (7.4%)	125 (60.4%)	
- Yes	25 (92.6%)	82 (39.6%)	
T Stage			**<0.001**
- T1	7 (25.9%)	86 (41.5%)	
- T2	13 (48.1%)	118 (57.0%)	
- T3	7 (25.9%)	3 (1.4%)	
N Stage			**<0.001**
- N0	2 (7.4%)	125 (60.4%)	
- N1	17 (63.0%)	79 (38.2%)	
- N2	6 (22.2%)	3 (1.4%)	
- N3	2 (7.4%)	0 (0.0%)	
Menopause Status			1.000
- No	16 (59.3%)	125 (60.4%)	
- Yes	11 (40.7%)	82 (39.6%)	
Laterality (1=Left, 2=Right)			0.161
- 1	9 (33.3%)	103 (49.8%)	
- 2	18 (66.7%)	104 (50.2%)	
Lymphovascular Invasion (LVI)			1.000
- No	18 (66.7%)	140 (67.6%)	
- Yes	9 (33.3%)	67 (32.4%)	
Estrogen Receptor (ER)			1.000
- Positive	19 (70.4%)	149 (72.0%)	
- Negative	8 (29.6%)	58 (28.0%)	
Progesterone Receptor (PR)			0.143
- Positive	13 (48.1%)	134 (64.7%)	
- Negative	14 (51.9%)	73 (35.3%)	
HER2 Status			0.675
- Positive	12 (44.4%)	79 (38.2%)	
- Negative	15 (55.6%)	128 (61.8%)	
Hormone Receptor (HR)			0.230
- Positive	26 (96.3%)	178 (86.0%)	
- Negative	1 (3.7%)	29 (14.0%)	
Endocrine Therapy			0.230
- No	1 (3.7%)	29 (14.0%)	
- Yes	26 (96.3%)	178 (86.0%)	
Immediate Reconstruction			0.803
- No	27 (100.0%)	201 (97.1%)	
- Yes	0 (0.0%)	6 (2.9%)	

Bold values indicate P<0.05.

The median follow-up duration for the entire cohort was 49.0 months (interquartile range [IQR], 30.2–70.0 months; range, 6.0–129.0 months). Over the course of the study period, 27 of the 234 patients (11.5%) developed breast cancer-related lymphedema (BCRL), translating to an overall incidence rate of 2.73 per 100 person-years. Among patients who developed BCRL, the median follow-up time was 37.0 months (IQR, 25.0–58.0 months), compared to 50.0 months (IQR, 31.8–71.0 months) for those who did not develop the condition.

### 3.2 Feature selection using LASSO regression

To develop a parsimonious and clinically interpretable prediction model, a two-step feature selection strategy was applied. First, LASSO-based variable screening was performed using L1-penalized logistic regression implemented in the scikit-learn library in Python. This step was used as an initial dimensionality-reduction procedure to limit potential overfitting given the relatively small number of outcome events. The regularization parameter was selected objectively by 5-fold cross-validation from a predefined grid of candidate values, and the optimal value in the final analysis was C = 0.76095. Variables with non-zero coefficients were retained as candidate predictors for subsequent modeling.

Second, predictors identified through LASSO screening, together with variables considered clinically important *a priori*, were entered into the multivariable logistic regression model. Final predictors were determined on the basis of both statistical significance and clinical relevance and were then used to construct the predictive nomogram. In the LASSO screening step, breast-conserving surgery showed the largest negative coefficient, whereas pectoral nodes dissection, N stage, and the number of harvested lymph nodes had relatively large positive coefficients, suggesting stronger contributions to BCRL risk stratification (**Table 2**). Results from the multivariable analysis are presented as odds ratios (ORs) with corresponding 95% confidence intervals (CIs).

**Table 2:** Initial feature screening using LASSO regression.

Selected feature	Coefficient (β)
Breast-conserving surgery	-4.287
Pectoral nodes dissection	2.931
N stage	2.214
Number of harvested lymph nodes	1.486
Total drainage volume (ml)	0.624
BMI (kg/m²)	0.512
Surgery duration (min)	0.401
Age (years)	0.366
Tumor size (mm)	0.329
HER2 (negative)	-0.284
Ki-67 index (%)	-0.173

The variables ultimately retained in the nomogram were also clinically coherent and represented the dominant risk domains identified by LASSO screening, namely surgical extent and nodal disease burden. Although several additional variables had non-zero LASSO coefficients, the final predictors were prioritized because they showed stronger coefficient magnitudes, clearer clinical interpretability, and more stable associations in the subsequent Firth penalized logistic regression model.

### 3.3 Multivariable analysis using Firth penalized logistic regression

Although the LASSO screening step identified a broader set of candidate predictors, only six variables were carried forward into the multivariable logistic regression analysis: surgery type, pectoral nodes dissection, number of harvested lymph nodes, N stage, tumor size, and BMI. These variables were selected because they were retained after LASSO screening, were clinically interpretable and readily available in routine practice, and represented the most relevant domains of BCRL risk, including treatment extent, nodal burden, tumor burden, and patient-related characteristics. To avoid unnecessary model complexity and reduce the risk of overfitting given the limited number of outcome events, variables with substantial conceptual overlap or weaker clinical relevance were not simultaneously retained in the multivariable model. This approach allowed the final model-building process to balance statistical parsimony with clinical applicability.

The six preselected candidate predictors were further evaluated using Firth penalized logistic regression, including surgery type, pectoral nodes dissection, number of harvested lymph nodes, N stage, tumor size, and BMI (**Table 3**). Among these variables, breast-conserving surgery remained independently associated with a substantially lower risk of BCRL compared with mastectomy (OR = 0.020, 95% CI: 0.002–0.161, P < 0.001). In contrast, pectoral nodes dissection (OR = 17.11, 95% CI: 1.48–197.26, P = 0.023), a greater number of harvested lymph nodes (OR = 1.13, 95% CI: 1.02–1.25, P = 0.021), and higher N stage (OR = 6.67, 95% CI: 1.52–29.34, P = 0.012) were independently associated with increased BCRL risk. BMI showed a borderline positive association with BCRL (OR = 1.39, 95% CI: 0.97–1.99, P = 0.075), whereas tumor size did not remain statistically significant after adjustment (OR = 1.04, 95% CI: 0.99–1.09, P = 0.132).

**Table 3:** Firth penalized logistic regression analysis of six candidate predictors for breast cancer-related lymphedema.

Variable	Coefficient	OR	95% CI Lower	95% CI Upper	P value
Breast-conserving surgery	-3.92307	0.01978	0.002426	0.161266	0.000248
Pectoral nodes dissection	2.839898	17.11401	1.484825	197.2552	0.02279
Number of harvested lymph nodes	0.11971	1.12717	1.018049	1.247987	0.021204
N stage	1.898211	6.673945	1.518259	29.33725	0.011979
Tumor size (mm)	0.037763	1.038485	0.988744	1.090728	0.131564
BMI (kg/m²)	0.328194	1.388458	0.967909	1.991733	0.074616

OR, odds ratio; CI, confidence interval.

Firth penalized logistic regression was used to reduce sparse-data bias and improve the stability of effect estimates in the setting of a limited number of outcome events.

Accordingly, the final prediction model and nomogram were constructed using the four predictors that remained statistically significant in the Firth model: surgery type, pectoral nodes dissection, number of harvested lymph nodes, and N stage.

### 3.4 Development of the predictive nomogram

**Figure 1:**
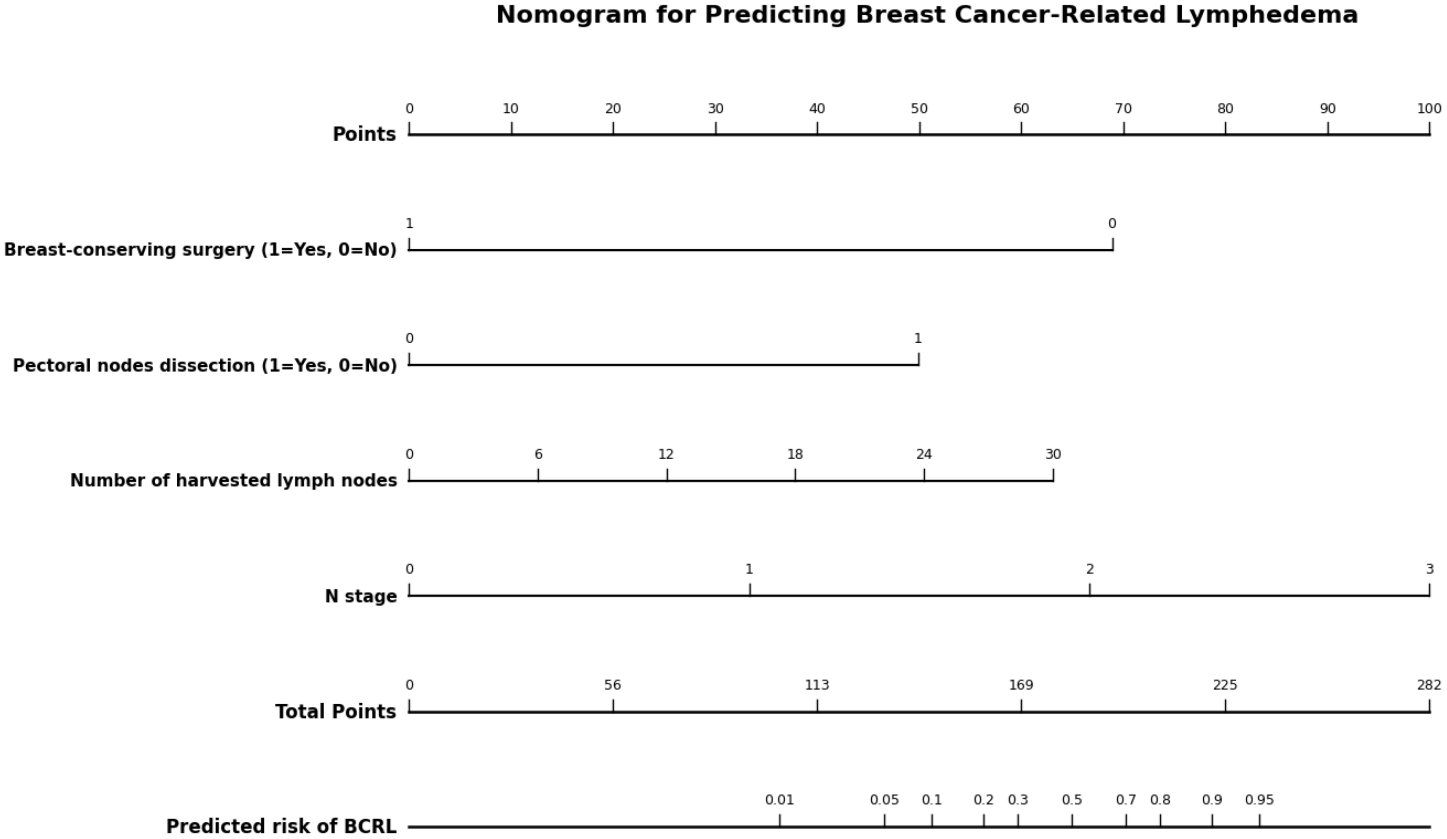
Nomogram for predicting the risk of breast cancer-related lymphedema.

To balance statistical parsimony with clinical applicability, a predictive nomogram was developed to estimate the individualized risk of breast cancer-related lymphedema (BCRL). Candidate predictors were initially screened using LASSO-based regression and subsequently evaluated using Firth penalized logistic regression. The final predictive model retained four clinically meaningful variables: surgery type, pectoral nodes dissection, number of harvested lymph nodes, and N stage. These four predictors were incorporated into the nomogram to provide an intuitive graphical tool for individualized postoperative risk estimation (**Figure 1**).

To use the nomogram, the clinician identifies the patient’s value for each predictor and draws a vertical line upward to the “Points” axis to determine the corresponding score. The scores for all predictors are then summed to obtain the “Total Points,” which are projected downward to the bottom scale to estimate the predicted probability of BCRL. In this model, treatment extent and nodal burden contributed most strongly to the total score. Specifically, mastectomy, pectoral nodes dissection, higher N stage, and more extensive lymph node harvesting were associated with higher point totals and, consequently, a greater predicted risk of BCRL. By contrast, breast-conserving surgery contributed fewer points and was associated with a lower predicted risk. Overall, the nomogram indicates that patients with more extensive surgery and heavier nodal involvement are at the highest risk of postoperative lymphedema.

### 3.5 Validation and performance of the nomogram

**Figure 2:**
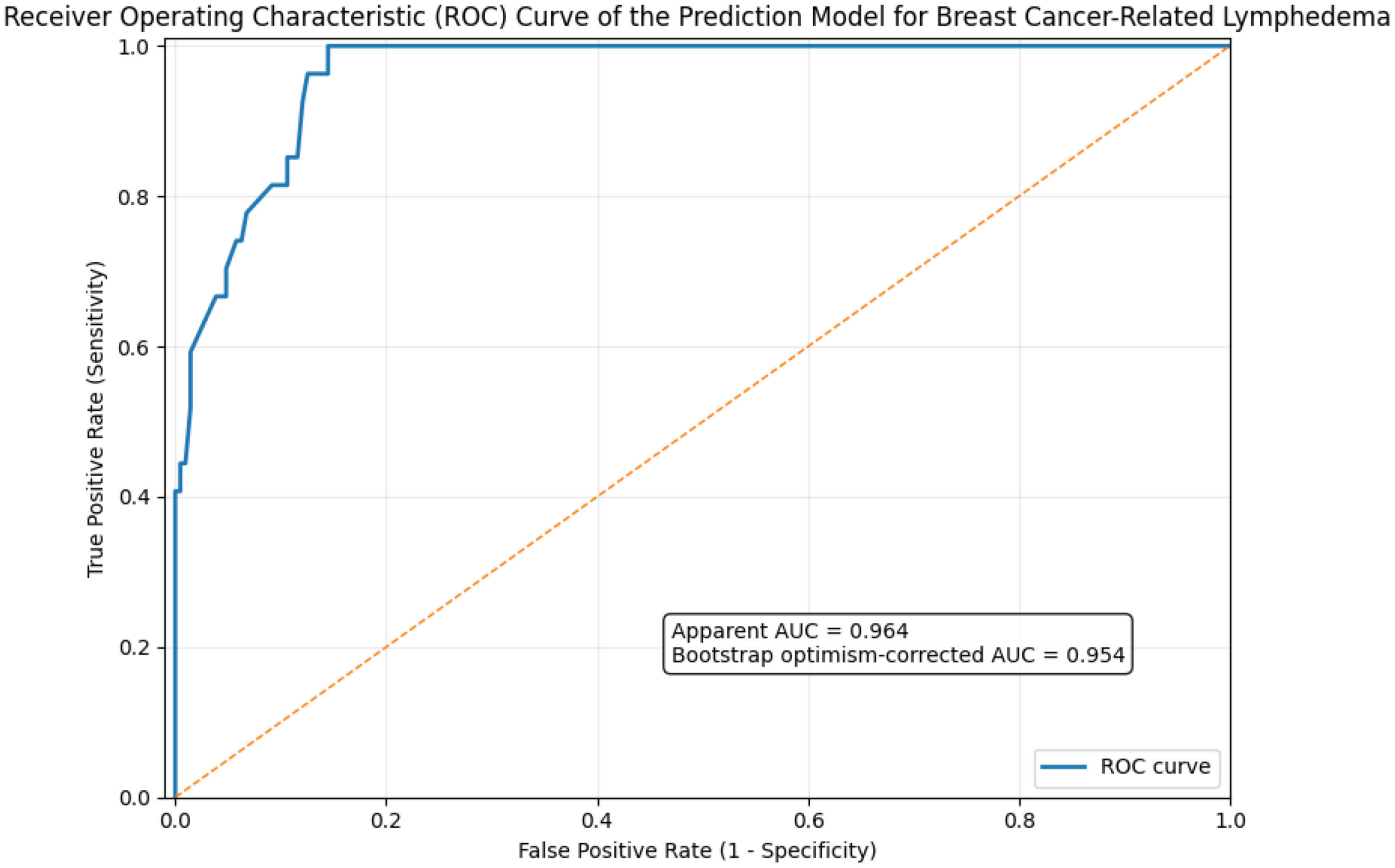
Receiver operating characteristic (ROC) curve of the prediction model for breast cancer-related lymphedema. The apparent AUC in the development dataset was 0.964, and the bootstrap optimism-corrected AUC was 0.954.

The discrimination performance of the nomogram was evaluated using receiver operating characteristic (ROC) curve analysis and bootstrap internal validation. As shown in **Figure 2**, the ROC curve demonstrated excellent discriminative ability, with an apparent AUC of 0.964. After internal validation using the bootstrap method, the optimism-corrected AUC was 0.954, indicating that the model retained strong discrimination after adjustment for potential overfitting.

Internal validation was performed with 1000 planned bootstrap resamples. Among these, 838 resamples were successfully fitted and included in the optimism estimation, whereas the remaining resamples were excluded because of model non-convergence during repeated fitting. The estimated mean optimism was 0.0105, suggesting that the apparent model performance was only minimally inflated. The final model was developed in 234 patients, including 27 BCRL events and 207 non-events.

Overall, these findings indicate that the nomogram has excellent discriminatory performance and only limited optimism, supporting the robustness of the model for individualized risk prediction of breast cancer-related lymphedema.

**Figure 3:**
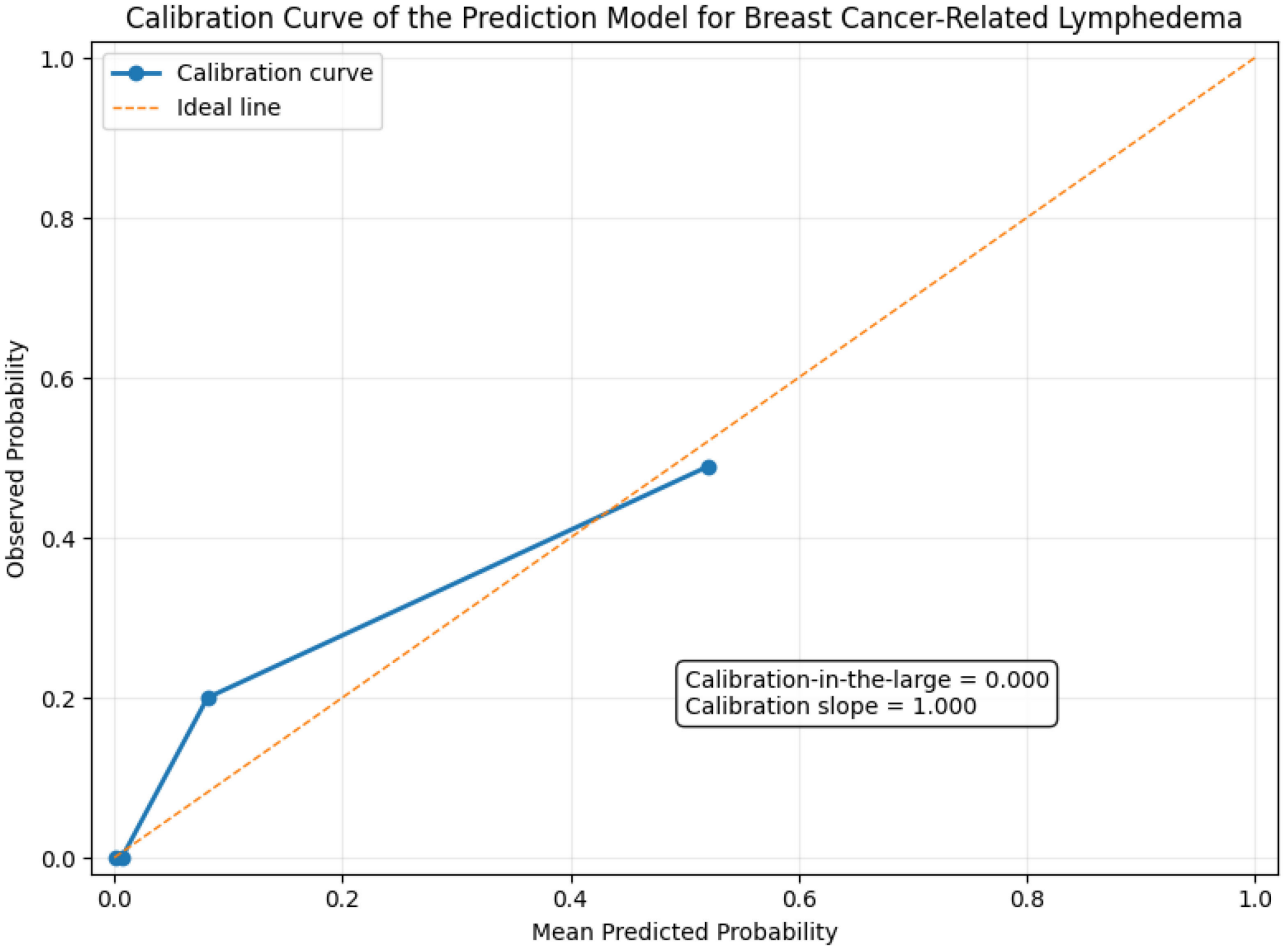
Apparent calibration curve of the prediction model for breast cancer-related lymphedema in the development dataset. Bootstrap internal validation showed an optimism-corrected calibration slope of 0.711, indicating residual overfitting.

The apparent calibration performance of the prediction model was further assessed using calibration-in-the-large, calibration slope, Brier score, and calibration curve analysis. (**Figure 3**) In the development dataset, the model showed an apparent calibration-in-the-large of 0.000 and an apparent calibration slope of 1.000, suggesting no obvious systematic overestimation or underestimation of risk in the apparent analysis. The apparent Brier score was 0.0469, supporting good overall predictive accuracy in the development dataset. Bootstrap-corrected calibration metrics are reported separately in **Table 4** to distinguish apparent performance from optimism-corrected performance.

**Table 4:** Apparent, optimism, and optimism-corrected performance of the prediction model based on bootstrap internal validation.

Metric	Apparent performance	Optimism	Optimism-corrected performance
AUC	0.964	0.011	0.954
Calibration slope	1.000	0.289	0.711
Brier score	0.0469	-0.0069	0.0537

Internal validation was performed using 1000 planned bootstrap resamples, of which 838 yielded valid model fits and were included in the final optimism estimation. Apparent performance refers to model performance estimated directly in the development dataset. Optimism-corrected performance was calculated by subtracting the estimated optimism from the apparent performance and provides a more conservative estimate of expected model performance in new but similar patients.

### 3.6 Bootstrap internal validation and optimism-corrected performance

Further bootstrap-based internal validation showed that the apparent calibration slope was 1.000 and the optimism-corrected calibration slope was 0.711, suggesting some degree of overfitting. Importantly, the optimism-corrected calibration slope of 0.711 indicates residual overfitting despite the use of penalized regression and bootstrap internal validation. A calibration slope below 1.0 suggests that the model coefficients may be somewhat overestimated and that predicted probabilities may be too extreme when the model is applied to new patients. Therefore, although the optimism-corrected AUC remained high, the predicted absolute risks generated by the nomogram should be interpreted with caution. Future external validation should evaluate whether global shrinkage, recalibration, or model updating is necessary before routine clinical implementation. The apparent Brier score was 0.0469, and the optimism-corrected Brier score was 0.0537, indicating acceptable overall predictive accuracy. (**Table 4**).

### 3.7 Decision curve analysis

**Figure 4:**
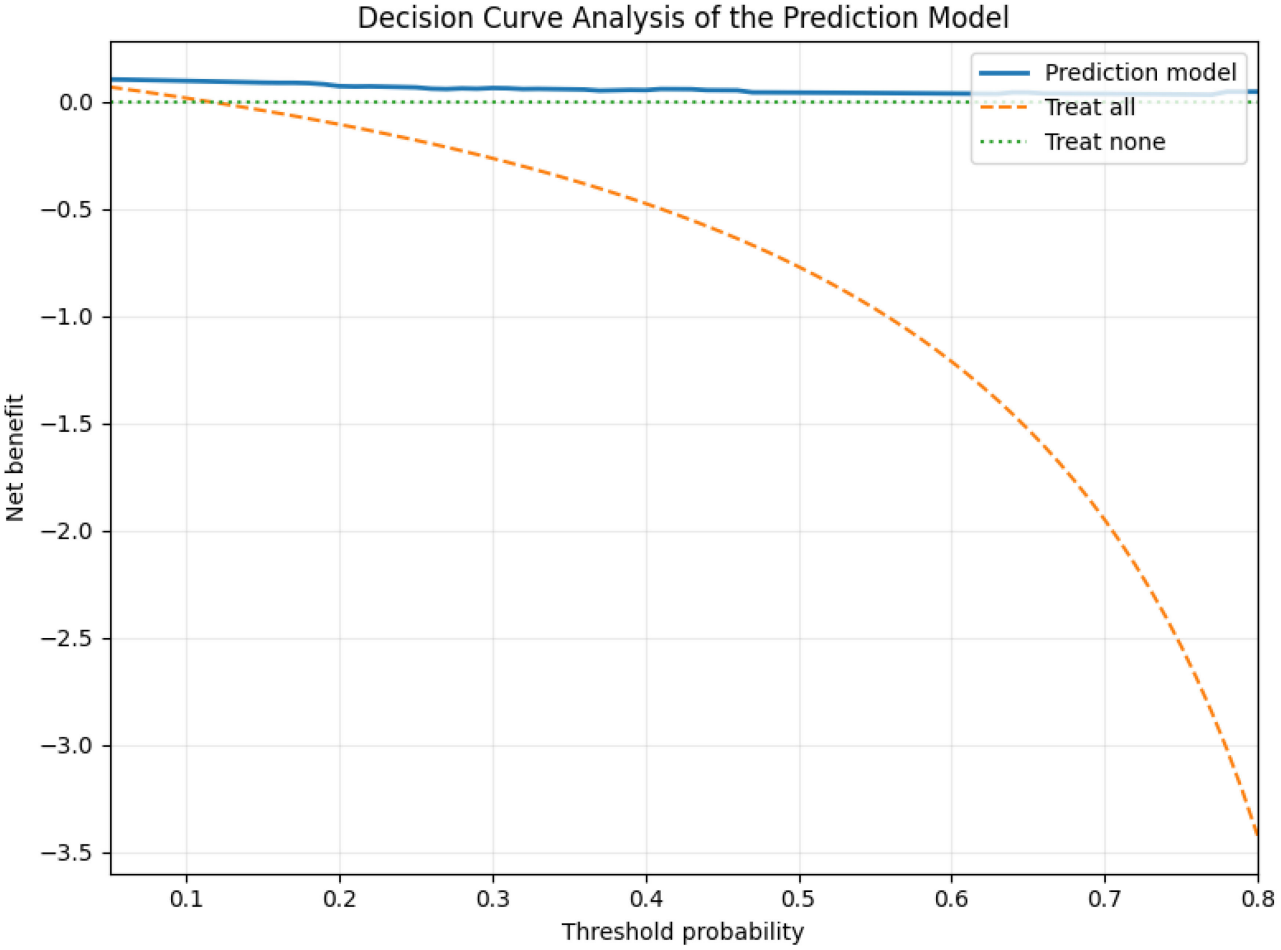
Decision curve analysis (DCA) for the BCRL predictive nomogram.

DCA was performed to evaluate the potential clinical utility of the prediction model across a threshold probability range of 5% to 80% (**Figure 4**). Within this range, the model generally provided a higher net benefit than the default strategies of treating all patients or treating none, supporting its potential value in clinical decision-making. Lower threshold probabilities represent more aggressive preventive strategies, in which clinicians may choose to intervene even when the estimated risk of BCRL is relatively low. In contrast, higher threshold probabilities correspond to more selective, resource-intensive intervention strategies reserved for patients at particularly high predicted risk. Thus, the selected threshold range was intended to cover a broad spectrum of clinical decision scenarios, from conservative to more proactive management approaches.

### 3.8 Missing data

Missing data were limited to two continuous variables: total drainage volume (34/234, 14.53%) and surgery duration (7/234, 2.99%). No missing data were observed in the outcome variable or in the key clinicopathological predictors retained in the final model. In the primary analysis, missing values were handled using median imputation. To further evaluate whether the study findings were sensitive to the method used for handling missing data, we performed a sensitivity analysis using multiple imputation by chained equations (MICE). Ten imputed datasets (m = 10) were generated, with 20 iterations performed for each imputation chain. All available study variables, including the outcome and relevant clinicopathological covariates, were incorporated into the imputation model to improve the plausibility of the missing-at-random assumption and to preserve the overall data structure. The same regression model was fitted separately in each of the 10 imputed datasets, and the regression coefficients and standard errors were pooled using Rubin’s rules to derive pooled odds ratios, 95% confidence intervals, and P values. This sensitivity analysis was used primarily to evaluate the robustness of predictor–outcome associations under an alternative missing-data strategy. The pooled estimates were materially similar to those obtained from the median-imputation analysis, particularly for the main predictors related to surgical extent and nodal burden. Model performance metrics were not used as the primary basis for inference in the multiple-imputation analysis, although no major qualitative inconsistency in model behavior was observed. The results of the multiple-imputation sensitivity analysis were materially similar to those of the primary analysis, indicating that the main findings were robust to the missing-data handling strategy. Effect estimates were broadly consistent between the median-imputation analysis and the multiple-imputation sensitivity analysis (**Supplementary Table 2**; **Supplementary Figure 1**).

## 4 Discussion

Accurate prediction of BCRL is essential for identifying high-risk patients, guiding early preventive interventions, and improving postoperative quality of life among breast cancer survivors. In the present study, we developed and internally validated a clinically practical nomogram for predicting ipsilateral upper-limb BCRL after breast cancer surgery. Using a revised modeling strategy that combined LASSO-based screening, Firth penalized logistic regression, and bootstrap internal validation, we identified four clinically meaningful predictors that were ultimately retained in the final nomogram: surgery type, pectoral nodes dissection, number of harvested lymph nodes, and N stage. The resulting model showed excellent apparent discrimination and retained strong performance after bootstrap optimism correction, together with acceptable calibration and favorable clinical utility across a broad range of decision thresholds.

A key methodological strength of the present study lies in the use of a staged modeling strategy to address the challenges of a relatively small-event dataset. With only 27 BCRL events, conventional multivariable regression using a large initial set of candidate predictors would have been vulnerable to overfitting and unstable effect estimation. To reduce dimensionality, we first applied LASSO-based screening using L1-penalized logistic regression with cross-validated tuning of the regularization parameter. We then carried forward a smaller set of clinically relevant candidate variables into Firth penalized logistic regression, which was specifically chosen to reduce sparse-data bias and improve coefficient stability in the setting of limited events. This approach yielded more conservative and clinically plausible effect estimates than the original conventional logistic regression model and provided a more robust basis for nomogram construction.

Clinically, our findings emphasize that treatment extent and nodal burden are the principal drivers of postoperative BCRL risk. Breast-conserving surgery was associated with a substantially lower risk of BCRL, whereas pectoral nodes dissection, higher N stage, and a greater number of harvested lymph nodes were associated with increased risk. This pattern is broadly consistent with previous literature showing that more extensive axillary intervention and greater nodal disease burden are major determinants of lymphedema after breast cancer treatment (2, 8, 9). In particular, our findings support the importance of interpectoral or pectoral node dissection. Anatomically, interpectoral nodes may function as part of collateral lymphatic drainage pathways, and disruption of this region may further compromise upper-extremity lymphatic outflow when primary axillary channels have already been disturbed. This is consistent with prior reports suggesting that interpectoral dissection may contribute to more severe or more frequent lymphedema (9, 10).

Our study also aligns with broader evidence that nodal burden is a major contributor to BCRL risk. The independent association of higher N stage with postoperative lymphedema is biologically plausible, because greater nodal involvement often accompanies more extensive regional surgery and reflects heavier locoregional disease burden. Similarly, the number of harvested lymph nodes likely captures the cumulative extent of lymphatic disruption. These findings reinforce the concept that BCRL is not driven by a single perioperative factor, but rather by the combined effects of disease extent and treatment intensity.

Compared with the original conventional logistic regression results, the revised Firth penalized logistic regression yielded substantially more conservative effect estimates. This is important because the very large odds ratios observed in the initial model suggested possible sparse-data bias and quasi-complete separation. Under such conditions, some degree of coefficient inflation cannot be excluded, and effect sizes derived from standard maximum-likelihood estimation may appear overly extreme. The subsequent Firth analysis preserved the overall direction of association while reducing the magnitude of the estimates, supporting the robustness of the identified predictors while also indicating that the original odds ratios should be interpreted with caution. This issue is particularly relevant for calibration and clinical interpretability, because inflated coefficients can lead to overly extreme predicted probabilities and may reduce the plausibility of a bedside risk tool.

The performance assessment of the present nomogram was also strengthened in the revised analysis. The model showed excellent apparent discrimination, with only limited optimism in AUC after bootstrap correction. However, the optimism-corrected calibration slope was 0.711, indicating residual overfitting and suggesting that the fitted regression coefficients may still be too large for direct transport to new populations. In practical terms, this means that the model may generate predicted probabilities that are overly extreme, particularly for patients at very low or very high estimated risk. Therefore, although the model appears useful for risk stratification, its absolute risk estimates should be interpreted cautiously until external validation is performed. Future studies should assess whether coefficient shrinkage, recalibration, or model updating is needed to improve calibration in independent cohorts. Decision curve analysis further indicated that the nomogram may provide clinical net benefit over the treat-all and treat-none strategies across a threshold range of 5 percent to 80 percent. Lower threshold probabilities correspond to more proactive preventive management, whereas higher thresholds reflect reserving more resource-intensive interventions for patients at particularly high predicted risk. This range was chosen to reflect a broad spectrum of plausible clinical decision scenarios rather than a single universal treatment cutoff.

Our findings are generally concordant with prior evidence, but some discrepancies should also be acknowledged. Variables such as BMI and other treatment-related factors have been identified in previous studies as contributors to BCRL risk (11, 12). In our dataset, BMI showed only a borderline association and was not retained in the final nomogram. Likewise, some treatment-related variables often discussed in the literature did not emerge as dominant predictors in the final model. These differences may reflect the limited number of BCRL events, local treatment practices, incomplete availability of highly granular treatment parameters, and residual instability inherent to small-sample modeling. Accordingly, our model should be understood as a clinically pragmatic prediction tool developed from the variables available in this institutional dataset, rather than as an exhaustive representation of every possible BCRL risk determinant.

Several limitations should be acknowledged. First, this was a retrospective, single-center study, and selection bias cannot be excluded. The lack of external validation limits the generalizability of the model across institutions and populations. Second, the relatively small number of BCRL events increases the risk of overfitting, unstable coefficient estimates, and limited transportability (13). Although we attempted to mitigate these issues through LASSO-based screening, restriction of candidate predictors, Firth penalized logistic regression, and bootstrap internal validation, residual instability cannot be fully excluded. Third, omitted variable bias and residual confounding remain possible. Although clinically relevant predictors were considered during model development, several established BCRL-related treatment factors could not be included in sufficient detail. In particular, granular radiotherapy parameters, such as regional nodal irradiation fields, dose-volume information, fractionation schedules, and boost details, as well as regimen-specific chemotherapy information, including taxane exposure, cumulative dose, number of cycles, and treatment sequence, were not consistently documented across the retrospective study period. Their exclusion may have resulted in residual confounding. It is also possible that some of their effects were partially captured by correlated variables such as N stage, surgery type, and the number of harvested lymph nodes. Future prospective studies should collect these treatment-related variables in a standardized manner to improve causal interpretation and model transportability. Fourth, BCRL was defined using circumference-derived volume calculations based on routinely collected follow-up data. Although measurements were originally obtained during routine postoperative follow-up using a standardized clinical protocol, circumference-derived volume remains an indirect method and may be affected by measurement variability, inter-observer differences, and some degree of non-differential outcome misclassification. Fifth, the model was developed using a single feature-selection and modeling framework. Although the final predictors were clinically plausible and remained directionally consistent across LASSO screening and Firth penalized regression, formal stability selection using repeated subsampling, bootstrapped variable selection frequencies, or extensive alternative penalty specifications was not performed. This may introduce model-selection bias, and future studies should examine predictor robustness using stability selection, ensemble feature-selection strategies, or independent validation datasets. Recent lymphedema prediction studies have suggested that interpretable machine learning and ensemble approaches may improve predictor robustness in some settings (14). Finally, although bootstrap resampling was used for internal validation, the model was not evaluated using temporal validation or external validation. Because the cohort spanned a long study period, future studies should examine transportability across time using temporally separated and independent validation datasets. Given the absence of external validation, the present nomogram should be used cautiously in clinical practice. At this stage, it should be regarded as a supportive tool for preliminary risk stratification within settings similar to the development cohort, rather than as a stand-alone instrument for definitive clinical decision-making. The model may be useful for identifying patients who may benefit from closer postoperative surveillance, patient education, early rehabilitation referral, or preventive counseling. However, it should not be used to mandate or withhold specific interventions without consideration of clinical judgment, patient preference, and institutional practice. External validation and, if necessary, recalibration are required before broader implementation.

Future work should therefore focus on prospective, multicenter validation in larger and more diverse cohorts. Such studies should incorporate more granular treatment-related variables, including radiotherapy dosimetry, chemotherapy subtype information, and possibly longitudinal BMI trajectories. Additional work may also compare the present nomogram with alternative modeling approaches, including ensemble feature-selection methods and other machine learning algorithms, to determine whether predictive performance and robustness can be improved without sacrificing interpretability. Integration of the model into electronic health records or digital risk calculators may further enhance its practical value in routine postoperative surveillance.

In summary, we developed and internally validated a clinically practical nomogram for predicting BCRL after breast cancer surgery. Using a revised modeling framework that combined LASSO-based screening, Firth penalized logistic regression, and bootstrap internal validation, we identified four key predictors: surgery type, pectoral nodes dissection, number of harvested lymph nodes, and N stage. The model demonstrated strong discrimination, acceptable overall predictive accuracy, and potential clinical utility. However, the optimism-corrected calibration slope indicated residual overfitting, and the absence of external validation limits immediate clinical implementation. Therefore, the nomogram should currently be used only as a supportive risk-stratification tool to guide closer surveillance and preventive counseling, rather than as a stand-alone decision-making instrument. Future external and temporal validation studies are needed to assess transportability, calibration, and the potential need for model shrinkage or recalibration.

## Data Availability

The original contributions presented in the study are included in the article/**Supplementary Material**. Further inquiries can be directed to the corresponding author.

## Supplementary material

Supplementary Table 1 Summary of missing data by variable.

Supplementary Table 2 Sensitivity analysis comparing median imputation and multiple imputation.

Supplementary Figure 1 Comparison of odds ratios between the median-imputation analysis and the multiple-imputation sensitivity analysis. Points indicate odds ratios and horizontal lines indicate 95% confidence intervals. The main predictors, including mastectomy, pectoral nodes dissection, and number of harvested lymph nodes, remained stable across the two missing-data strategies, whereas the association for total drainage volume was attenuated after multiple imputation.

## References

[1] Jørgensen MG Toyserkani NM Hansen FG Bygum A Sørensen JA . The impact of lymphedema on health-related quality of life up to 10 years after breast cancer treatment. NPJ Breast Cancer. (2021) 7:70. doi: 10.1038/s41523-021-00276-y 34075045 PMC8169644

[2] DiSipio T Rye S Newman B Hayes S . Incidence of unilateral arm lymphoedema after breast cancer: a systematics review and meta-analysis. Lancet Oncol. (2013) 14:500–15. doi: 10.1016/S1470-2045(13)70076-7. PMID: 23540561

[3] International Society of Lymphology . The diagnosis and treatment of peripheral lymphedema: 2023 consensus document of the International Society of Lymphology. Lymphology. (2023) 56:133–51. doi: 10.2458/lymph.5632 39207406

[4] Norman SA Localio AR Kallan MJ Weber AL Torpey LMS Potashnik B . Lymphedema in breast cancer survivors: incidence, degree, time course, treatment, and symptoms. J Clin Oncol. (2009) 27:390–7. doi: 10.1200/JCO.2008.17.9291. PMID: 19064976 PMC2645852

[5] Shen A Qiang W Zhang L Wang Q Xu H Lu T . Risk factors for breast cancer-related lymphedema: an umbrella review. Ann Surg Oncol. (2024) 31:284–302. doi: 10.1245/s10434-023-14277-7. PMID: 37725224

[6] Lin Q Yang T Jin Y Tian T He J Lin Z . Prediction models for breast cancer-related lymphedema: a systematic review and critical appraisal. Syst Rev. (2022) 11:217. doi: 10.1186/s13643-022-02084-2. PMID: 36229876 PMC9559764

[7] Collins GS Moons KGM Dhiman P Riley RD Beam AL Van Calster B . TRIPOD+AI statement: updated guidance for reporting clinical prediction models that use regression or machine learning methods. BMJ. (2024) 385:e078378. doi: 10.1136/bmj-2023-078378. PMID: 38626948 PMC11019967

[8] Gillespie TC Sayegh HE Brunelle CL Daniell KM Taghian AG . Breast cancer-related lymphedema: risk factors, precautionary measures, and treatments. Gland Surg. (2018) 7:379–403. doi: 10.21037/gs.2017.11.04. PMID: 30175055 PMC6107585

[9] Liu X Sun K Yang H Xia L Lu K Meng X . Risk factors for the development of severe breast cancer-related lymphedema: a retrospective cohort study. BMC Cancer. (2023) 23:361. doi: 10.1186/s12885-023-10814-5. PMID: 37081431 PMC10116791

[10] García-Vilanova Comas A García Vilanova A Fuster-Diana E Martínez-Alzamora N Fernández-Tena J García-Vilanova Comas J . Prognostic value of the interpectoral lymph nodes in breast cancer. A 20-year survival study. Clin Transl Oncol. (2006) 8:108–18. doi: 10.1007/s12094-006-0167-9. PMID: 16632425

[11] Ugur S Arıcı C Yaprak M Mescı A Arıcı GA Dolay K . Risk factors of breast cancer-related lymphedema. Lymphat Res Biol. (2013) 11:72–5. doi: 10.1089/lrb.2013.0004. PMID: 23772716 PMC3685313

[12] Kwan ML Darbinian J Schmitz KH Citron R Partee P Kutner SE . Risk factors for lymphedema in a prospective breast cancer survivorship study: the Pathways Study. Arch Surg. (2010) 145:1055–63. doi: 10.1001/archsurg.2010.231. PMID: 21079093 PMC2997775

[13] van Smeden M Moons KG de Groot JA Collins GS Altman DG Eijkemans MJ . Sample size for binary logistic prediction models: beyond events per variable criteria. Stat Methods Med Res. (2019) 28:2455–74. doi: 10.1177/0962280218784726. PMID: . Epub 2018 Jul 3. 29966490 PMC6710621

[14] Teo PT Rogacki K Gopalakrishnan M Das IJ Abazeed ME Mittal BB . Determining risk and predictors of head and neck cancer treatment-related lymphedema: a clinicopathologic and dosimetric data mining approach using interpretable machine learning and ensemble feature selection. Clin Transl Radiat Oncol. (2024) 46:100747. doi: 10.1016/j.ctro.2024.100747. PMID: 38450218 PMC10915511

