# Supplementary material for: Development and internal validation of a risk prediction model for ipsilateral upper-limb lymphedema following breast cancer surgery
Source: Front Oncol. 2026 Jun 3;16:1823165. doi: 10.3389/fonc.2026.1823165 (PMC13271979; doi:10.3389/fonc.2026.1823165)
Supplement: Supplementary Table 1 — Summary of missing data by variable. [file Table1.docx]

Supplementary Table S1. Summary of missing data by variable

| **Variable Name** | **Missing Count** | **Missing Percentage (%)** | **Data Type** |
| --- | --- | --- | --- |
| **Total Drainage (ml)** | 34 | 14.53% | Continuous |
| **Surgery Duration (min)** | 7 | 2.99% | Continuous |
| Breast Cancer-Related Lymphedema (BCRL) | 0 | 0.00% | Categorical |
| Axillary Management | 0 | 0.00% | Categorical |
| Interpectoral Lymph Node Dissection | 0 | 0.00% | Categorical |
| Chemotherapy | 0 | 0.00% | Categorical |
| Age | 0 | 0.00% | Continuous |
| BMI | 0 | 0.00% | Continuous |
| Menopausal Status | 0 | 0.00% | Categorical |
| T stage / N stage | 0 | 0.00% | Ordinal |
| Nodes Harvested | 0 | 0.00% | Continuous |
| Radiotherapy | 0 | 0.00% | Categorical |
| Endocrine Therapy | 0 | 0.00% | Categorical |
| ... (Other Variables) | 0 | 0.00% | - |
