# Supplementary material for: Development and internal validation of a risk prediction model for ipsilateral upper-limb lymphedema following breast cancer surgery
Source: Front Oncol. 2026 Jun 3;16:1823165. doi: 10.3389/fonc.2026.1823165 (PMC13271979; doi:10.3389/fonc.2026.1823165)
Supplement: Supplementary Table 2 — Sensitivity analysis comparing median imputation and multiple imputation. [file Table2.docx]

Supplementary Table S2. Sensitivity analysis comparing median imputation and multiple imputation

| **Variable** | **Median imputation OR (95% CI)** | **P value** | **Multiple imputation pooled OR (95% CI)** | **P value** |
| --- | --- | --- | --- | --- |
| Mastectomy | 123.584 (7.520–2030.975) | 0.001 | 110.985 (6.892–1787.337) | 0.001 |
| Pectoral nodes dissection | 109.078 (6.329–1880.052) | 0.001 | 104.171 (6.381–1700.703) | 0.001 |
| Number of harvested lymph nodes | 1.193 (1.082–1.315) | <0.001 | 1.198 (1.086–1.322) | <0.001 |
| Number of positive lymph nodes | 1.407 (0.947–2.091) | 0.091 | 1.394 (0.935–2.078) | 0.103 |
| Tumor size | 0.999 (0.951–1.049) | 0.971 | 1.001 (0.954–1.050) | 0.960 |
| Surgery time | 1.027 (1.002–1.052) | 0.031 | 1.026 (1.002–1.051) | 0.033 |
| Total drainage volume | 1.005 (1.000–1.010) | 0.044 | 1.004 (0.999–1.009) | 0.116 |

**Abbreviations:** OR, odds ratio; CI, confidence interval.
**Note:** Multiple-imputation estimates were pooled across 10 imputed datasets using Rubin’s rules. The sensitivity analysis was based primarily on pooled regression coefficients and odds ratios, rather than on re-estimation of a separate final nomogram or pooled calibration curve.
