# Supplementary material for: Development and internal validation of a risk prediction model for ipsilateral upper-limb lymphedema following breast cancer surgery
Source: Front Oncol. 2026 Jun 3;16:1823165. doi: 10.3389/fonc.2026.1823165 (PMC13271979; doi:10.3389/fonc.2026.1823165)
Supplement: Supplementary Figure 1 — Comparison of odds ratios between the median-imputation analysis and the multiple-imputation sensitivity analysis. Points indicate odds ratios and horizontal lines indicate 95% confidence intervals. The main predictors, including mastectomy, pectoral nodes dissection, and number of harvested lymph nodes, remained stable across the two missing-data strategies, whereas the association for total drainage volume was attenuated after multiple imputation. [file Supplementaryfile1.docx]

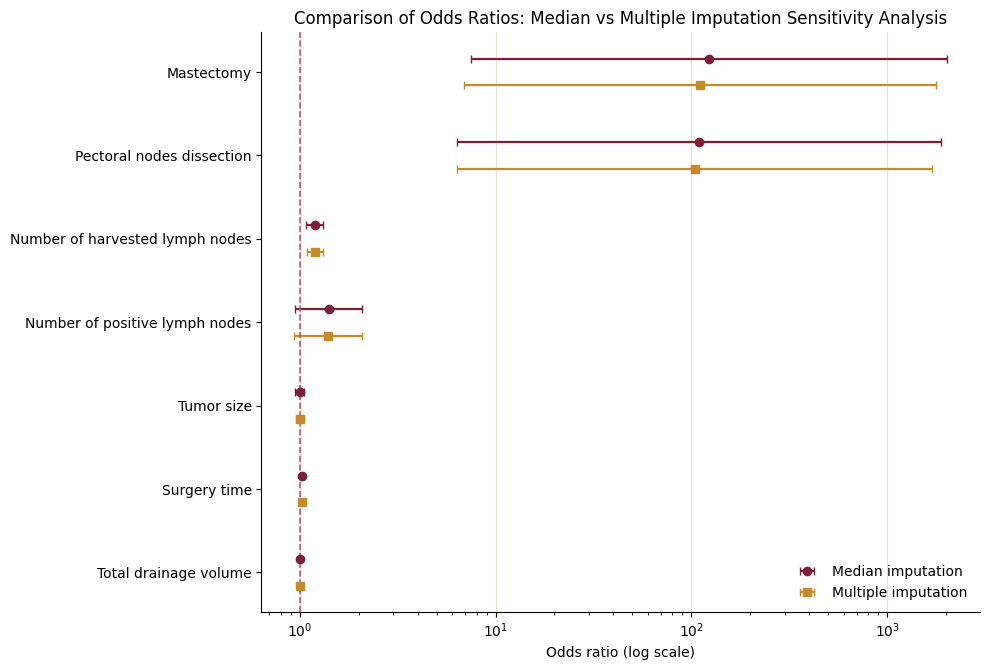


Supplementary Figure S1. Comparison of odds ratios between the median-imputation analysis and the multiple-imputation sensitivity analysis.

Points indicate odds ratios and horizontal lines indicate 95% confidence intervals. The main predictors, including mastectomy, pectoral nodes dissection, and number of harvested lymph nodes, remained stable across the two missing-data strategies, whereas the association for total drainage volume was attenuated after multiple imputation.
